# First Report of CC5-MRSA-IV-SCC*fus* “Maltese Clone” in Bat Guano

**DOI:** 10.3390/microorganisms9112264

**Published:** 2021-10-31

**Authors:** Assia Mairi, Abdelaziz Touati, Alix Pantel, Alex Yahiaoui Martinez, Mourad Ahmim, Albert Sotto, Catherine Dunyach-Remy, Jean-Philippe Lavigne

**Affiliations:** 1 Laboratoire d’Ecologie Microbienne, Faculté des Sciences de la Nature et de la Vie, Université de Bejaia, Bejaia 06000, Algeria; assia-mairi@hotmail.fr (A.M.); ziz1999@yahoo.fr (A.T.); 2 Bacterial Virulence and Chronic Infection, Institut National de la Santé et de la Recherche Médicale U1047, University Montpellier, 30908 Nîmes, France; 3 Bacterial Virulence and Chronic Infection, Institut National de la Santé et de la Recherche Médicale U1047, Department of Microbiology and Hospital Hygiene, University Montpellier, Nîmes University Hospital, 30029 Nîmes, France; alix.pantel@chu-nimes.fr (A.P.); alex.yahiaouimartinez@chu-nimes.fr (A.Y.M.); catherine.remy@chu-nimes.fr (C.D.-R.); 4 Laboratoire d’Ecologie et d’Environnement, Faculté des Sciences de la Nature et de la Vie, Université de Bejaia, Bejaia 06000, Algeria; forestecolo@gmail.com; 5 Bacterial Virulence and Chronic Infection, Institut National de la Santé et de la Recherche Médicale U1047, Department of Infectious and Tropical Diseases, University Montpellier, Nîmes University Hospital, 30029 Nîmes, France; albert.sotto@chu-nimes.fr

**Keywords:** Algeria, bat guano, Maltese clone, toxic shock syndrome toxin, ST149

## Abstract

Methicillin-resistant *Staphylococcus aureus* (MRSA) is a widespread pathogen that could cause different illnesses in both human and animals. Presence of MRSA in animals raises concerns of their capacity to act as reservoirs, particularly in wild animals. This study aimed to characterize the resistance and virulence patterns of *S.* *aureus* strains isolated from bat guano in Algeria. From March to May 2016, 98 bat guano samples from Aokas’s cave (Bejaia, Algeria) were collected. Swabs were taken for microbiological studies. Isolates were identified by Vitek^®^ MS system, and antibiotic susceptibility was determined by disk diffusion method. The clonal origin, virulence and antibiotic resistance profiles of *S. aureus* isolates were characterized by whole genome sequencing. Eleven *S.* *aureus* strains were obtained from the 98 guano samples. Seven isolates were sensitive to all antibiotics tested and four (36.3%) were resistant to penicillin G, cefoxitin and fusidic acid. The four MRSA isolates were assigned to the sequence type ST149 and related to *spa* type *t*010. These isolates harbored a SCC*mec*IV element and the fusidic acid resistance element Q6GD50 (*fusC*). They carried different virulence genes including several enterotoxins (*sea*, *egc* enterotoxin locus, *sec*, *sel*), and the toxic shock syndrome toxin (*tst*). Our results highlight that bat guano may constitute an important reservoir of MRSA strains.

## 1. Introduction

*Staphylococcus aureus* can colonize and/or infect human and animals and this bacterium is considered as one of the most serious public health threats [1,2,3]. In hospital, *S. aureus* represents a frequently occurring pathogen isolated in various samples, and it is also the second most common pathogen in the community [4]. Methicillin-resistant *S. aureus* (MRSA) has emerged and diffused rapidly in hospitals just after the introduction of penicillin M [4,5]. To date, this MRSA is one of the most important nosocomial pathogens, causing high rates of morbidity and mortality worldwide [1,4]. Subsequently, a set of predominant clones has been characterized. Currently, ST5 and ST239, USA300, and ST398 are the frequently encountered clones in the healthcare-associated MRSA (HA-MRSA), the community-associated MRSA (CA-MRSA) and the livestock-associated MRSA (LA-MRSA), respectively [4,5].

The expression of different virulence factors, such as the staphylococcal enterotoxins, toxic shock syndrome toxin-1 (TSST-1), hemolysins, and Panton–Valentine leukocidin (PVL) is the main cause of the infectious capacity of *S. aureus* and its success as pathogen [6]. Some MRSA clones have been found to harbor these toxinogenic markers. In parallel, MRSA has emerged in different reservoirs, and is currently disseminated in the community and has been frequently found to colonize or infect animals [3]. Indeed, MRSA has been discovered in pets, livestock, and wild animals [7,8].

Bats are one of the most widespread and diversified mammalian groups, with more than 1300 species, living in a wide variety of habitats and climatic zones [9,10]. They play an important role as pollinators of economically important plants and could be used as a source of animal protein [11]. However, bats have also received increasing attention as potential reservoir and vectors of zoonotic microorganisms such as Ebola virus, coronavirus and Marburg virus [12,13]. In addition to the various viruses that have been identified in bats, bacteria of clinical importance have also been described [14,15]. Among the bacteria, *S. aureus* has been detected in bats [16,17,18,19,20,21], mainly described in Sub-Saharan African regions. However, the presence of MRSA has rarely been reported [21,22]. In 2007, these strains were first isolated from feces collected from diverse bats in Poland and 23% of staphylococci were methicillin resistant comprising MRSA and *Staphylococcus sciuri* [22]. In 2020, a MRSA strain belonging to the CC152 and harboring the PVL was described in different fomites samples (e.g., currency note, computer keyboard, cell phone) in Obafemi Awolowo University, Nigeria with a possible transmission from fruit bats (*Eidolon helvum*) [21].

In Algeria, the prevalence of MRSA is of concern notably due to the diffusion of the ‘European’ ST80-IV CA-MRSA PVL+ clone [4]. Recently, our team highlighted the diffusion of toxinogenic MRSA in different ecological niches (humans, pets, livestock, wildlife, food and aquatic environment), suggesting a wildlife MRSA reservoir in this country [8].

The objectives of this study were to: (1) evaluate the presence of *S. aureus* and MRSA in bat guano in Aokas’s cave (Bejaia, Algeria) and (2) characterize the resistome and virulome of the MRSA isolates. This work provides evidence that the multidrug resistance (MDR) is now widespread in a country where the prevalence of MDR bacteria is high. Wild animals represent a reservoir that contributes to the dissemination and transfer of the resistance genes.

## 2. Materials and Methods

### 2.1. Fecal Samples

**Figure 1:**
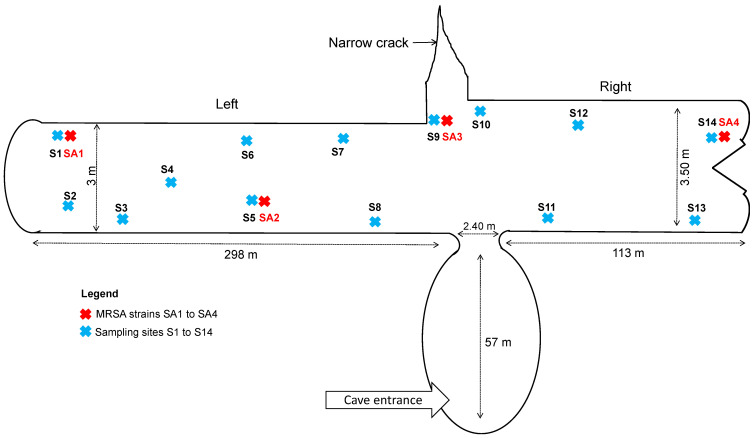
Schematic representation of the Aokas’s cave. Sx, site of sampling (x, number); SAx, site of *S. aureus* isolation (x, number).

Between March and May 2016, bat guano samples from *Microchiroptera* were obtained from Aokas’s cave (located 27 km East of Bejaia city, Algeria). Bats present in caves belong to troglophilic species including *Asellia tridens*, *Rhinolophus blasii*, *Rhinolophus clivosus*, *Rhinolophus euryale*, *Rhinolophus ferrumequinum*, *Rhinolophus hipposideros*, *Rhinolophus mehelyi*, *Rhinopoma hardwickii*, *Myotis punicus*, *Myotis capaccinii Myotis emarginatus*, *Plecotus gaisleri*, *Taphozous nudiventris* and *Miniopterus schreibersii*. These species overwinter in caves, and during the summer (and the breeding season) they seek warmer shelters (e.g., attics, roofs of houses and mosques) and are in proximity to humans. Aokas’s cave is an artificial tunnel that has an entrance in the form of a large natural spherical chamber, with a 113 m long tunnel on the right side, which leads into the open air through a narrow opening. It is 3.5 m wide and its height varies from 2 m to 2.7 m. On the left side is a tunnel of 298 m length, 3 m width and 2 m height. Between the two tunnels, there is a natural narrow crack facing the entrance, perpendicular to the tunnel (Figure 1). This cave is known to shelter bats only. Collection plates (using plastic petri dish with rising edge) were disposed the day before the sampling in the different sites of collection (entry (*n* = 3), center (*n* = 5) and far end (*n* = 6) of the cave). These plates avoided contamination because the guano were not on the cave floor and no insects could reach the fecal pellets and contaminate it. Fresh pellets were collected (14 samplings in 7 periods) in the plates, early in the morning. They were mixed using sterile swabs (Copan, Brescia, Italy). All samples were analyzed within the day after sampling in the Microbiological Ecology Laboratory at the University of Bejaia (Algeria).

Swabs were inoculated in 1mL of Trypticase Soy Broth (TSB) (Fluka, St. Louis, MO, USA) containing colistin (10 mg/L), aztreonam (10 mg/L) and amphotericin B (2 mg/L). After 24h incubation at 37 °C, a 200 μL aliquot was steaked onto mannitol salt agar plates and incubated for 48 h at 37 °C.

In total, 98 fecal samples were collected. However, due to the sampling method used, we were unable to assign one fecal sample to individual bats. To avoid multiple isolates from one bat, we exclusively included one isolate per sampling site and date in the final analysis.

### 2.2. Bacterial Identification

Bacterial identification was performed on all colonies grown on plates using the Vitek^®^ MS system (bioMérieux, Marcy l’Etoile, France).

### 2.3. Antimicrobial Susceptibility Testing

Antimicrobial susceptibility was determined by the disk diffusion method (BioRad, Marnes La Coquette, France) on Mueller–Hinton agar plates according to European Committee on Antimicrobial Susceptibility Testing (EUCAST) 2019 [23].

The following antibiotics were tested: penicillin G (1 μg), cefoxitin (30 μg), erythromycin (15 μg), clindamycin (2 μg), quinupristin/dalfopristin (15 μg), kanamycin (30 μg), tobramycin (10 μg), gentamicin (10 μg), minocycline (30 μg), ofloxacin (5 μg), fusidic acid (10 μg), fosfomycin (200 μg), rifampicin (5 μg) and cotrimoxazole (1.25–23.75 μg). The minimum inhibitory concentration (MIC) values of isolates to vancomycin and teicoplanin were determined by microbroth dilution (Umic^®^, Biocentric, Bandol, France) over a range of dilutions (0.25–4 mg/L for vancomycin and 0.25–8 mg/L for teicoplanin). Resistance to methicillin was evaluated using cefoxitin disks. The detection of *mecA* and *mecC* genes was performed by PCR as described [24].

### 2.4. Whole-Genome Analysis

The four MRSA isolates were sequenced. The strains were grown aerobically on 5% Columbia sheep blood agar plates (Becton Dickinson, Le Pont-de-Claix, France) at 37 °C for 24 h. Genomic DNA was extracted by EZ1 DNA Tissue Kit (QIAGEN, Courtaboeuf, France). Whole Genome Sequencing (WGS) was performed with an Illumina MiSeq sequencing system (Illumina, San Diego, CA, USA) using the paired-end (PE) read libraries (PE250) prepared by Nextera XT DNA Library Prep Kit (Illumina) following the manufacturer’s protocol. Raw reads were processed using FastQC (v.0.11.7) to assess data quality. The Cutadapter tool (v.1.16) implemented in Python (v.3.5.2) was used to remove residual PCR primers and filter low quality bases (Q_score < 30) and short reads (<150 bp). The filtered trimmed reads were included in the downstream analysis. Obtained reads were mapped against *S. aureus* NCTC 8325 genome (GenBank accession number: GCA_000013425.1), employing the CLC genomics workbench 7 (Qiagen Inc., Valencia, CA, USA), using default parameters; length fraction: 0.5, similarity fraction: 0.8. The assembled contigs were processed by Prokka software for microbial genome annotation [25]. Virulence factor database (VFDB) (https://www.mgc.ac.cn/VFs/, accessed on 26 May 2021) was used to infer virulence factor-encoding genes from genome sequences [26]. Antimicrobial resistance genes were obtained from ABRIcate with the ResFinder database on assembled genomes [27,28]. The two whole genomes sequences (WGS) were aligned using the MAFFT software [29]. SNP calls were made from the PE library raw reads. For SNP analysis, we employed the following software: SNP-sites for variants calling [30] and SnpEff (v.1.3T) for SNP annotation in coding regions [31]. SNP annotations of affected genes were searched within wild-type genome and their effects were classified according to mutation impact. Genes affected by stop gain mutations were searched in Uniprot database for virulence classification. Additional analyses were performed on WGS such as circular genome representation (BLAST Ring Image Generator software (BRIG) [32]). Raw reads of newly sequenced MRSA strains are available under the BioProject number PRJNA734263 and assembled genomes SAMN19487653, SAMN19487654 and SAMN19487655.

## 3. Results

A total of 11 *S. aureus* isolates were studied. Seven *S. aureus* isolates were sensitive to all antibiotic tested. The four remaining isolates were resistant to penicillin G, cefoxitin and fusidic acid (Table S1). PCR amplification showed that these four isolates were positive for *mecA* gene. No *mecC* gene was detected.

Only the four MRSA isolates were submitted to whole genome sequencing to determine their genetic determinants. The genome sizes obtained varied between 2,734,323 to 2,795,689 bp with a high similarity (>99% identity) (Table 1). The four genomes exhibited a similar GC content around 32.7%. The numbers of coding DNA sequences (CDS) were 2498 for B-AoC-SA2, 2539 for B-AoC-SA1, 2541 for B-AoC-SA4 and 2556 for B-AoC-SA3.

**Figure 2:**
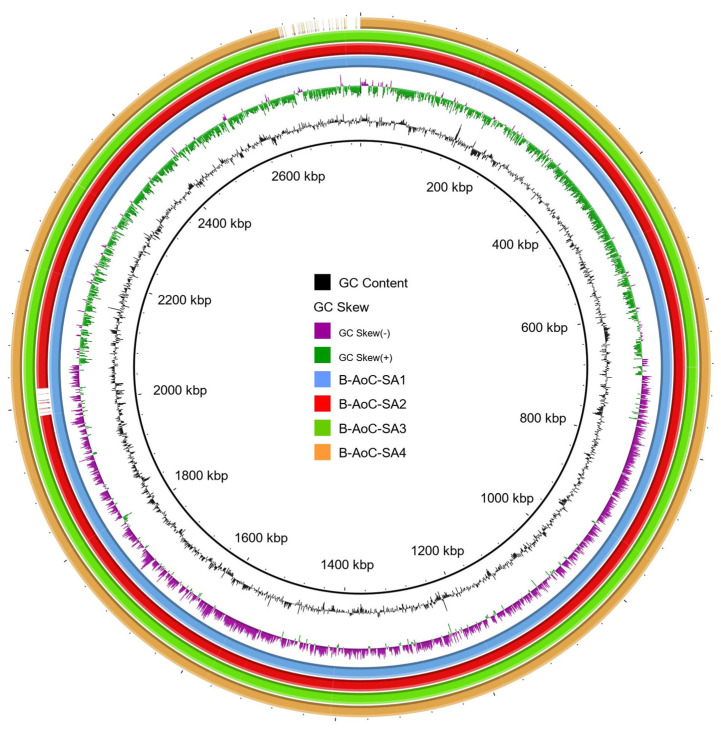
BRIG analysis of MRSA genomes isolated from bat guano. Genomes of B-AoC-SA2 (red ring), 3 (green ring), 4 (orange ring) isolates were compared against the genome of B-AoC-SA1 (blue ring) isolate using BRIG software [32]. Only regions with >90% nucleotide identity are colored. Lower identity percentage or no match are represented by blank spaces in each ring.

The circularized maps of the *S. aureus* strains based on the predicted CDS showed that both genomes exhibited the same overall organization (Figure 2). Each genome presented 61 rRNA. The four MRSA strains were assigned to the ST149, termed the “Maltese clone” and related to the *spa* type *t*010. They also harbored the SCC*mec*IV element (Table 2).

These isolates carried the putative fusidic acid resistance element, Q6GD50 (*fusC*), which was correlated with phenotypic resistance to fusidic acid. They also harbored *fosB* (coding for fosfomycin resistance) and *blaZ* (coding for penicillinase) genes. For the virulome profile, the whole genome analysis showed that all strains carried the toxic shock syndrome toxin- (*tst*), enterotoxins- (*sea*, *sec*, *sel*, *egc*-cluster), leukocidins- (*lukF*, *lukS*, *lukD*, *lukE* and *lukY*), adhesion factors- (belonging to the microbial surface components recognizing adhesive matrix molecules) (*clfA*, *fnbA*, *fnbB*, *fib*, *map*, *eno*, *bbp*, *ebh*, *ebpS*, *sasG*, *sdrC*, *sdrD* and *vwb*), and hemolysins-encoding genes (*hl*, *hla*, *hllll*, *hlb* and *hlgA*). The isolates belonged to the *agr* type II and harbored the *cap5* gene (Table 2). These isolates harbored neither exfoliative toxins-encoding genes (*eta*, *etb*, *etd*), nor epithelial cell differentiation inhibitors (*edinA*, *edinB*, and *edinC*) and *lukF/S-PV* genes. The genome analysis confirmed that the four isolates were closely related according to Ankrum et al.’s criteria [33]. Variant call analysis showed the presence of SNPs within coding regions, comparing the four *S. aureus* isolates. When evaluating SNPs in B-AoC-SA1 strain compared to the three other strains, 58 divergent nucleotides were identified, and affected 2.3% of the genes (58/2539). Among these differences, 10 SNPs concerned nonsense (Stop gain) mutations affecting genes that were classified according to their functions (virulence factor-encoding genes/other genes). These mutations directly affected one well-known virulence factor (*clfB* (clumping factor B-encoding gene) in B-AoC-SA1) and other non-virulence factor (*hin* (DNA-invertase encoding gene), *bacC* (oxydase-encoding gene), *rnmV* (ribonuclease-encoding gene), *gloB* (gluthatione hydrolase-encoding gene), *fabG3* (3-oxoacyl-reductase encoding gene), *ugpQ* (glycerol-phosphodiesterase-encoding gene), *lon* (ATP-dependent protease-encoding gene), *paaZ* (phenylacetic aid-coenzyme A encoding gene) and *recD* (helicase-encoding gene).

## 4. Discussion

MRSA isolates are one of the main pathogens causing worldwide public health problems. They have disseminated, not only in the hospital setting, but also in the community. MRSA have also been reported among various animal species including companion animals, livestock, and wild animals in several countries [3]. In Algeria, the data on MRSA have been reported from patients admitted to hospital, while insufficient studies have so far been obtained regarding genotypes of MRSA strains isolated outside hospitals [34,35,36], especially from wild animals [8,37,38].

Thus, we report in this study the first isolation of MRSA from bat guano in Algeria. The four MRSA isolates recovered in this study belonged to the same clone, ST149-MRSA-IV-SCC*fus*, which was described for the first time in Malta [39,40]. It was also reported more recently in the United Arab Emirates [41]. Interestingly, this clone has never before been detected in extra-human niches. Moreover, the description of MRSA in bats is very rare [21,22]. We also observed that our isolates harbored *tst* gene. The presence of this toxinogenic marker in wild animals has been previously described in wild monkeys and apes [42], wild rabbits [43], red deer [44] and white storks [45]. It is noteworthy that these toxinogenic *S. aureus* strains are described in wild animals, mainly in regions where these strains have high prevalence in clinical settings. For example, PVL+ strains have been previously isolated from wildlife in Algeria [8] in a context of high rates among isolates from human [46]. The same trend was reported in Nigeria, where MSSA-PVL+ [19] and MRSA-PVL+ [21] isolates have been described in this PVL-endemic region. The *tst* gene was also highly described in Algeria in clinical settings [47] and in livestock [37], wildlife [8] and food products [48]. Altogether, these results suggest that bats could be a reservoir of toxinogenic *S. aureus* strains. Moreover, a recent study at the Aokas’s cave reported the presence of carbapenemase-producing Enterobacterales (KPC- and OXA-48-producing *Klebsiella pneumoniae*) in bat guano [49]. This additional result suggests that the bat guano could serve as reservoir of MDR bacteria.

The origin of the MRSA isolates remains an important question. Several studies highlighted that water could be a source of dissemination in nature [50,51,52]. Besides the microbiota diversity in the gastrointestinal tract, the presence of bacteria and their antibiotic-resistance patterns could be also influenced by dietary habits of bats [53]. Notably, Algerian bat species are insectivorous [54]. The ingestion of insects could be an additional route of transmission of antibiotic-resistant bacteria [55]. Moreover, this situation is mainly related to the overuse of antibiotics and their existence in soil and wastewater [56], the low disinfection of sewage resulting in the bacterial contamination of wastewater treatment plant and the insufficient infection control measures. The colonization of wild animals with MRSA lineages has been noticed from animals in close contact with humans [57]. However, the direct mode of transmission remains unclear between bat guano and humans. Besides, *S. aureus* clones adapted to animals might undergo further host-adaptive evolutionary changes, favoring an epidemic spread of new and more virulent strains in the human population and vice versa. Two factors associated with *S. aureus* immune evasion determinants in humans (*scn* and *sak*) were present in our isolates. They are responsible for the human innate immune system modulation and are considered as adaptation mechanisms of *S. aureus* inside the human host [58]. Their presence suggests the possible human origin of these isolates.

The presence of MRSA strains from wildlife, especially from bats, is cause for alarm, with implications for animal reservoirs of resistant bacteria [59]. Interestingly, we identified that the four isolates harbored the *fosB* gene, whereas they were not resistant to fosfomycin. This resistance can occur by emergence of chromosomal mutations and/or the acquisition by horizontal genes transfer of plasmid-bearing enzymes that inactivate fosfomycin. It has previously been described that mutations in *murA* (affecting the MurA affinity of fosfomycin), and *glpT* and *uhpT* genes (encoding fosfomycin transport systems of bacteria) are more common and important to generate fosfomycin-resistant strains [60]. Moreover, fosfomycin activity can also be supplied by catalytic activity such as FosA, FosB, FosC and FosX [61,62,63]. Due to the longevity of bats (35 years), the detection of these MDR bacteria could explain the ability of bats to maintain these MRSA, develop persistent carriage and transmit them to animals or human by direct or indirect contamination (via droplets, mucus, saliva or feces produced by bats).

The major limitation of our study was the impossibility to associate isolates with individual bats and to estimate the prevalence of bats colonized by MRSA. However, we applied rigorous inclusion criteria to rule out a sampling bias. The four MRSA isolated in our study were obtained at different periods (although these periods all fell over spring) on fresh fecal pellets collected on swabs in different parts of the cave. Bats are a protected species in Algeria, thus sampling methods had to be limited to guano collection or samples performed on recently dead animals.

## 5. Conclusions

Bats represent reservoir hosts of bacteria of significance for human health. These microorganisms can cross the species barrier to infect humans and animals. Studies on the importance of bats as reservoir of microorganisms are rare. This study highlighted that bat guano could be considered as a source of MRSA in Algeria. Their presence in a secluded area reinforces the extreme diversification of the diffusion of MRSA isolates. This wide dissemination outside of the clinical setting may represent an emerging risk for public health.

## Figures and Tables

**Table 1:** General genome characteristics of MRSA strains isolated from bat guano.

Features	B-AoC-SA1	B-AoC-SA2	B-AoC-SA3	B-AoC-SA4
Genome size (bp)	2,777,157	2,734,323	2,795,689	2,776,793
Genome Coverage	64.36X	85.14X	72.08X	60.20X
Contigs	35	46	60	34
G + C Content (%)	32.67	32.70	32.67	32.67
CDS	2539	2498	2556	2541
tRNA	61	61	61	61
MLST	149	149	149	149
Locality	Bejaia (Algeria)	Bejaia (Algeria)	Bejaia (Algeria)	Bejaia (Algeria)

**Table 2:** Characteristics of MRSA isolated from bat guano in Aokas’s cave (Bejaia, Algeria).

Strain Type	Strain	Date Isolated and Isolation Site ^1^	Antimicrobial Resistance Phenotype ^2^	Antibiotic Resistance Genes	Specific Toxinogenic Profile	Leucocidins Genes	Enterotoxins Genes ^3^	Hemolysins Genes	MSCRAMMs Genes	Immune Evasion Genes
CC5-MRSA-IV (Maltese clone)/ ST149; *t*010, *agr* group II	B-AoC-SA1	17 March 2016 S1	PEN, FOX, FUS	*mecA, blaZ, blaI, blaR, fusC* (Q6GD50)	*tst1+, pvl-, etA-, etB-, etD-*	*lukF, lukS, lukD, lukE, lukY*	*sea, sec, sel, egc-*cluster	*hl, hla, hllll, hlb, hlgA*	*bbp, clfA, ebh, ebpS, eno, fib, fnbA, fnbB, map, sasG, sdrC, sdrD, vwb*	*sak, scn*
CC5-MRSA-IV (Maltese clone)/ ST149; *t*010, *agr* group II	B-AoC-SA2	1 April 2016 S5	PEN, FOX, FUS	*mecA, blaZ, blaI, blaR, fusC* (Q6GD50)	*tst1+, pvl-, etA-, etB-, etD-*	*lukF, lukS, lukD, lukE, lukY*	*sea, sec, sel, egc-*cluster	*hl, hla, hllll, hlb, hlgA*	*bbp, clfA, clfB, ebh, ebpS, eno, fib, fnbA, fnbB, map, sasG, sdrC, sdrD, vwb*	*sak, scn*
CC5-MRSA-IV (Maltese clone)/ ST149; *t*010, *agr* group II	B-AoC-SA3	16 April 2016 S9	PEN, FOX, FUS	*mecA, blaZ, blaI, blaR, fusC* (Q6GD50)	*tst1+, pvl-, etA-, etB-, etD-*	*lukF, lukS, lukD, lukE, lukY*	*sea, sec, sel, egc-*cluster	*hl, hla, hllll, hlb, hlgA*	*bbp, clfA, clfB, ebh, ebpS, eno, fib, fnbA, fnbB, map, sasG, sdrC, sdrD, vwb*	*sak, scn*
CC5-MRSA-IV (Maltese clone)/ ST149; *t*010, *agr* group II	B-AoC-SA4	17 May 2016 S14	PEN, FOX, FUS	*mecA, blaZ, blaI, blaR, fusC* (Q6GD50)	*tst1+, pvl-, etA-, etB-, etD-*	*lukF, lukS, lukD, lukE, lukY*	*sea, sec, sel, egc-*cluster	*hl, hla, hllll, hlb, hlgA*	*bbp, clfA, clfB, ebh, ebpS, eno, fib, fnbA, fnbB, map, sasG, sdrC, sdrD, vwb*	*sak, scn*

^1^ Isolation sites are available in Figure 1; ^2^ PEN, penicillin G, FOX, cefoxitin, FUS, fusidic acid; ^3^ *egc* cluster includes to *seg*, *sei*, *sem*, *sen* and *seo* genes.

## Data Availability

Raw reads of newly sequenced MRSA strains are available under the BioProject number PRJNA734263 and assembled genomes SAMN19487653, SAMN19487654 and SAMN19487655.

## Supplementary Materials

The following are available online at https://www.mdpi.com/article/10.3390/microorganisms9112264/s1, Table S1: Antibiotic susceptibility profile of *S. aureus* strains isolated from Aokas’s cave (Bejaia, Algeria).
