# Supplementary material for: First Report of CC5-MRSA-IV-SCCfus “Maltese Clone” in Bat Guano
Source: Microorganisms. 2021 Oct 31;9(11):2264. doi: 10.3390/microorganisms9112264 (PMC8619057; doi:10.3390/microorganisms9112264)
Supplement: Supplementary file 1 [file microorganisms-09-02264-s001.zip › microorganisms-1442275-supplementary.pdf]

Table S1. Antibiotic susceptibility profile of 11 *S. aureus* strains isolated from bat guano in Aokas's cave (Bejaia, Algeria).

**Antibiotics**	**Number of isolates (%) susceptible to antibiotics**
Penicillin G	4 (36%)
Cefoxitin	4 (36%)
Erythromycin	0 (0%)
Clindamycin	0 (0%)
Quinupristin/dalfopristin	0 (0%)
Kanamycin	0 (0%)
Tobramycin	0 (0%)
Gentamicin	0 (0%)
Minocycline	0 (0%)
Ofloxacin	0 (0%)
Fusidic acid	4 (36%)
Fosfomycin	0 (0%)
Rifampicin	0 (0%)
Cotrimoxazole	0 (0%)
Vancomycin	0 (0%)
Teicoplanin	0 (0%)
